# INITIAL EFFICACY OF THE SAVVY CAREGIVER PROGRAM IN KOREAN AMERICAN DEMENTIA CAREGIVERS

**DOI:** 10.1093/geroni/igad104.2548

**Published:** 2023-12-21

**Authors:** Yuri Jang, Kenneth Hepburn, Juyoung Park, William Haley, Miyong Kim

**Affiliations:** University of Southern California, Los Angeles, California, United States; Emory University, Atlanta, Georgia, United States; University of Southern California, Los Angeles, California, United States; University of South Florida, Tampa, Florida, United States; University of Texas at Austin, Austin, Texas, United States

## Abstract

Dementia caregivers in ethnic minority communities have often been excluded from access to caregiver interventions and their benefits because of linguistic and cultural barriers. In pursuit of equal treatments for all, it is imperative to make evidence-based interventions available and accessible to ethnic minority dementia caregivers, and linguistic and cultural adaptation is indispensable for reaching out to them. In the present study, we adapted the Savvy Caregiver Program for Korean American dementia caregivers and tested its initial efficacy for reducing depressive symptoms. This pilot intervention was delivered to 13 Korean American dementia caregivers by two Savvy-certified Korean-speaking trainers. Over the 6-week period of program implementation, there was no attrition (session completion rate = 98%). All participants’ depressive symptoms were reduced at completion of the program, and the level of reduction was statistically significant, with a large effect size (t = 8.64, p < .001, Cohen’s d = .89). Although patterns of change did vary among participants, the score difference between post-intervention and 6-month follow-up was not statistically significant (t = .74, p = .47). The program played a significant role in reducing depressive symptoms in this pilot sample of Korean American dementia caregivers, and this benefit was sustained at the 6-month follow-up. Along with the program’s notably high retention rate, its efficacy suggests strong unmet needs for psychoeducational programs among Korean American dementia caregivers. The study offers promise for sharing the therapeutic benefit of the Savvy program with linguistic minorities when delivered in a linguistically, culturally appropriate manner.

